# Describing the diurnal relationships between objectively measured mother and infant physical activity

**DOI:** 10.1186/s12966-018-0692-2

**Published:** 2018-06-25

**Authors:** Alessandra Prioreschi, Soren Brage, Kate Westgate, Lisa K. Micklesfield

**Affiliations:** 1 Department of Paediatrics, School of Clinical Medicine, Faculty of Health Sciences, MRC/WITS Developmental Pathways for Health Research Unit, University of Witwatersrand, Johannesburg, South Africa; 2 MRC Epidemiology Unit, University of Cambridge, Cambridge, UK

**Keywords:** Physical activity, Infant, Caregiving, Behaviour, Diurnal variation, Intensity distribution

## Abstract

**Background:**

Evidence for the importance of accumulating sufficient physical activity in the early years is mounting. This study aimed to determine the relationship between maternal and infant objectively measured physical activity, and to examine the diurnal interactions between these behaviours while accounting for potential covariates.

**Methods:**

Mothers and infants (*n* = 152 pairs; infants aged 3–24 months) were recruited from Soweto, South Africa, and physical activity was measured using a wrist worn accelerometer (Axivity AX3, Axivity Ltd., Newcastle-upon-Tyne, UK) for 3–7 days. Mothers completed sleep diaries recording night time-in-bed (used as a proxy for nocturnal sleep status) for themselves and their infant; and reported times during which their infant was in their personal care (caregiver status) for each day during the measurement period. Significant correlates of infant physical activity, as well as the interactions between mother’s physical activity, day of the week, sleep status, and caregiver status, were included in panel regression analyses with infant physical activity as the outcome.

**Results:**

There was an equal distribution of boys and girls, and their age ranged from 2.6 to 24.5 months. The majority of mothers (73%) did not spend any time apart from their infant. During weekdays, the combined effect of mother’s physical activity (β=0.11), the interactions between mother’s physical activity and caregiver status (β=0.17), and sleep status (β= − 0.04) on infant physical activity was β=0.24; while during weekend days this association was β=0.21; and was largely moderated by the interaction between the mother being with the infant and her activity levels (β=0.23), but partly attenuated by mother’s physical activity independent of other variables (β= − 0.04). For each hour of the day, for both mother and infant, peaks of physical activity were higher when the mother was not the primary caregiver.

**Conclusions:**

Infant physical activity levels were strongly associated with their mother’s activity levels particularly during the week; this relationship was stronger when mothers were more active while looking after their infant. Mothers should be encouraged to be active when looking after their children, particularly during the week, and to provide infants with as much opportunity to be active as possible.

## Background

Evidence for the importance of accumulating sufficient physical activity in the early years is mounting. Physical activity in the first few years of life has been associated with motor and cognitive development [1], and with body composition [1, 2]; and some tracking into later life has been shown [3, 4] therefore implying an association with metabolic risk in later life [5]. However, there is limited data available assessing physical activity in the first 2 years of life [6].

In the first 2 years of life, it is logical to assume that infant physical activity is largely dependent on their parents, and the opportunities that they provide their children to be active. Recommendations state that infants need to be provided with sufficient space to move and play, and that parents should provide a stimulating environment that encourages exploration and interaction, and that time spent restricted should be minimal thus allowing infants to move freely [7]. Movements in the first year of life often occur in sporadic, short-bursts [6], and should include a variety of activities such as arm and leg movements, tummy time (30 min per day is recommended) lifting of head, rolling, reaching, grasping, and eventually sitting and crawling. In the second year of life more defined movements such as walking and running, climbing and jumping start evolving; and recommendations advise 180 min of active time per day accumulated in a variety of ways and incorporating high energy activity [8, 9]. These movements are important for setting up motor competencies and physical literacy in early life. As children get older and their behaviours become more autonomous, it is likely that they will be influenced by their parents’ activity levels through role modeling, as well as through joint participation in physical activity [10, 11], and potentially even through a genetic predisposition to be more or less active [12, 13]. Indeed, studies have shown that physical activity levels tend to aggregate within families [14], particularly when considering very high and very low intensity activities [15–18]. Therefore, in order to improve infant physical activity, it seems justified to target their parents – who can be educated around the importance of providing opportunities for their infants to be active, can be provided with tools to increase their self-efficacy for effectively improving their infants’ physical activity, and can be advised to increase their own activity levels in order to improve their own health, as well as that of their infant. Indeed, existing behavioural interventions use parents as agents of change to affect multiple infant behaviours [19–22].

However, the evidence for associations between parent and child objectively measured physical activity levels is conflicted [23], and very little data exists examining these relationships in the first 2 years of life. This is an important period of life for setting up growth, health and behavioural trajectories, including establishing healthy physical activity patterns [4, 24]. There are various factors that have been shown to influence infant and young children’s physical activity levels and may thus influence the relationship between parent and child physical activity. For example, infant’s physical activity has been shown to be influenced by interaction with other children [25], developmental stage [26], and various maternal factors such as education, employment status and maternal activity behaviours [27–29]. Furthermore, diurnal variation in patterns of physical activity levels has been shown in toddlers [30, 31]. Since many children in Soweto do not have a father present in the home throughout the day [32], we focus here on maternal-infant interactions. Therefore, the aims of this study were to determine the relationship between maternal and infant objectively measured physical activity, and to examine the diurnal interactions between these behaviours while accounting for potential covariates.

## Methods

### Participants and procedures

For the purpose of this observational study, all infants (< 12 months) and toddlers (12–24 months) will be referred to as infants. Mothers and their infants (*n* = 152 pairs), who were already involved in a separate study at the Developmental Pathways for Health Research Unit (DPHRU) within the Chris Hani Baragwanath Academic Hospital, were contacted telephonically and invited to participate in this study. The recruitment strategy aimed to obtain a convenience sample with an equal spread of infants at various ages and developmental stages (i.e.: 3-, 6-, 12-, 18-, and 24-months). Exclusion criteria included any diagnosed developmental abnormalities that may impact on normal movement or development, as well as maternal inability to understand the questionnaires. Mothers were required to read and sign informed consent and assent documents for themselves and their infant, and were free to withdraw from the study at any time. Data collection took place at DPHRU in Soweto, South Africa. Soweto is a peri-urban setting, which is home to over 1.2 million inhabitants, accounting for one third of the population of Johannesburg (South Africa’s largest city). Ethical approval for this study was provided by the University of the Witwatersrand Human Research Ethics Committee (M150632). At the first visit, all demographic information was collected, anthropometric measurements taken, and both mother and infant were fitted with an accelerometer to be worn on their non-dominant wrist (all infants wore the monitors on their left wrist). Mothers were also provided with sleep- and caregiver diaries to complete. One week later, mothers were required to return the accelerometers and diaries. If participants were unable to return the devices, devices were collected from the participants’ homes.

### Measures

#### Mother and infant physical activity

Physical activity was measured using a wrist worn accelerometer (Axivity AX3, Axivity Ltd., Newcastle-upon-Tyne, UK), which has been used in large-scale adult population studies [33]. The accelerometer was worn in a standard silicon wrist-band by the mothers, and in a specially designed fabric band (Open Lab, Newcastle, UK) by the infants. The design and feasibility of this infant band has been described previously [34]; briefly the infant band was perceived to be safe, comfortable and acceptable according to mothers, and compliance wearing the band as well as technical reliability of the device data was very good (98% provided at least 3 days of valid data).

Monitors were initialised to capture triaxial acceleration data at 100 Hz with a dynamic range of + − 8 g. All participants were asked to wear the monitors at all times for a one-week period. Raw acceleration data was downloaded and auto-calibrated to local gravity using methods described elsewhere [35]. Vector magnitude was calculated as the root of sum of squared x-, y-, and z-axis acceleration, following which a high- (0.2 Hz) and low- (20 Hz) pass frequency filter was applied to the data in order to remove gravity, as well as high-frequency noise [36]. The resulting variable provides an approximation of acceleration due to human movement alone (expressed in mg). Physical activity was then summarised at 15-min level representing the average physical activity intensity for each time period [37]. Non-wear was identified based on the standard deviation of each axis being below 13 mg for > 1 h [37]. Since data was reported and compared at the 15-min interval level, any interval where non-wear was detected was excluded, and only periods with complete 15-min wear were included. Whole days were excluded if more than 40% of total accelerometer data for a given day was detected as non-wear. The accelerometer time-series data were annotated with maternal-reported night time-in-bed (nocturnal sleep status) for themselves and their infant, and with caregiver times according to the diaries (details below). Accelerometer time-series data were then coded with sleep status (yes/no) and caregiver status (yes/no).

#### Anthropometry and demographics

Mothers’ height was measured to the nearest 1 mm using a wall-mounted stadiometer (Holtain, UK), and weight was measured to the nearest 0.1 kg using a digital scale (Dismed, USA). Mothers’ body mass index (BMI) was calculated as (weight(kg)/height(m)^2^) and categorised as underweight (< 18.5 kg/m^2^), normal weight (≥18.5 kg/m^2^ and < 25 kg/m^2^) or overweight/obese (≥25.5 kg/m^2^). Infant length was measured to the nearest 1 mm using an infantometer (Chasmors Ltd., UK), and weight was measured to the nearest 0.1 kg using a digital scale (Dismed, USA). All anthropometry measurements were completed twice by trained research staff according to standardised procedures, and the average of the two values was used. Infant BMIs were converted to age-specific z scores according to the 2006/2007 World Health Organisation (WHO) growth standards [38] using the WHO Anthro software [39].

Mothers were asked to report their date of birth, and their infant’s date of birth and gender. Mothers were also asked to report whether they had any other children, as well as details of their employment (whether or not they had a full-time job and how many hours they worked per day during the week and on weekends).

### Diaries

During the measurement period (~ 7 days), mothers were asked to complete sleep diaries recording the time at which their infant was put to bed for the night, and the time at which they were picked up from bed in the morning. Mothers were also asked to report their own time-to-bed and wake times. If sleep diary data was missing, values were imputed based on sample trends for days and times from the whole sample. These time-in-bed variables were thus considered as crude proxies for nocturnal sleep status for mothers and infants. Mothers were further asked to report times during which their infant was not in their personal care for each day during the measurement period in order to determine caregiver status, and to describe who looked after their infant while not in their own care. Three mothers had missing caregiver data – two of these mothers had reported not having a full-time job and being full-time mothers, and were therefore considered to be with their infant at all times. The third mother reported having a job, and her reported working hours were therefore considered time apart from her infant, while non-work hours were considered time with her infant.

### Developmental milestones

According to maternal reported attainment of milestones, infants between 3 and 12 months were categorised as (1) not yet mobile (2) crawling or (3) walking. Between 12 and 18 months infants were categorised as (2) crawling or (3) walking, and at 24 months all infants were presumed to be walking [40].

### Statistical analysis

All statistical analyses were conducted using STATA 13 for Mac. Participant characteristics were summarised and presented as mean (SD), n (%), or median (IQR). Linear correlations were used to detect significant associations between infant and mother physical activity (mean vector magnitude, mg), and potential correlates thereof (infant gender, infant and maternal age, infant and maternal BMI, sleep status, caregiver status, duration apart, maternal employment and hours spent at her job, parity and number of siblings, and infant developmental stage). Significant correlates of infant physical activity (*p* < 0.05), as well as the interactions between mother’s physical activity, day of the week, sleep status, and caregiver status, were then included in panel regression analyses with infant physical activity as the dependent outcome. All regressions were controlled for maternal age and BMI, duration apart from infant, infant age and gender, infant BMI z score, developmental stage, number of siblings, maternal employment, and hour of the day. Data were stratified by week/weekend due to the significant independent effect of the week/weekend variable. Wilcoxon rank-sum tests were conducted to determine differences in infant physical activity levels according to whether mother and infant were together or not; these analyses were further stratified by age and gender. A *p* < 0.05 was considered significant in all cases.

## Results

Infant and mother characteristics are shown in Table 1. Two mother-infant pairs lost their devices, and a further three infants and four mothers lost their devices. One mother’s data was lost due to incorrect initialisation. Therefore, 142 mother-infant pairs provided useable physical activity data and were included in the analysis. There was an equal distribution of boys and girls, and their age ranged from 2.6 and 24.5 months, with a roughly equal distribution by age category: 22% were ≤ 3 months, 22% were between 3 and 6 months, 21% were between 6 and 12 months, 14% were between 12 and 18 months, and 21% were between 18 and 24 months. The majority of mothers (73%) did not spend any time apart from their infant (i.e.: they were the primary caregiver at all times) during the 7-day study period, and 27% of mothers were employed full-time during the study period. For those who did spend time apart from their infant, the mean duration of time apart was 8.3(3.9) hours per day. When mothers were not with their infant, 63% of infants were looked after by a friend or family member, 13% were in crèche, and 24% were looked after by a nanny. Two thirds of infants (67%) were not the first child, and the number of siblings (for those who were not single children) ranged from 1 to 5 (with a median of 1). For both mother and infant, physical activity was significantly higher on the weekend compared to during the week (Table 1, *p* < 0.01 for both mothers and infants).

**Table 1:** Participant characteristics

	Mean (SD) or Median (IQR) or n (%)
Infant factors	
Boys (n,%)	75 (53)
Age (months)	11.7 (2.6–24.5)
Infant BMI z-score	0.26 (1.67)
Physical activity	
Overall physical movement (mg)^a^	17 (5–64)
Physical movement on weekdays (mg)	16 (5–63)
Physical movement on weekend days (mg)	21 (5–67)
Developmental Stage	
Not yet mobile (n, %)	69 (49)
Crawling (n, %)	30 (21)
Walking (n, %)	43 (30)
Maternal factors	
Maternal age (years)	29 (6)
BMI (kg/m^2^)	28.3 (7.4)
Underweight (n, %)	7 (5)
Normal Weight (n, %)	45 (32)
Overweight/Obese (n, %)	90 (63)
Infant is first child (n, %)	47 (33)
Physical activity	
Overall physical movement (mg)	33 (6–78)
Physical movement on weekdays (mg)	31 (5–77)
Physical movement on weekend days (mg)	37 (6–79)

^a^Movement measures are median activity-related acceleration, summarised in 15-min intervals

Table 2 shows the results of the panel regression analyses between candidate exposures and infant physical activity (*n* = 142), stratified by week/weekend day. Mother’s physical activity was associated with infant physical activity regardless of the time of day, but this relationship was only significant on weekdays. During weekdays, the combined effect of mother’s physical activity (β=0.11), the interactions between mother’s physical activity and caregiver status (β=0.17), and sleep status (β= − 0.04), on infant physical activity was β=0.24; while during weekend days this association was β=0.21. On weekend days, this effect was largely moderated by the interaction between the mother being with the infant and her activity levels (β=0.23), but was partly attenuated by mother’s physical activity independent of other variables (β= − 0.04).

**Table 2:** Panel regression associations with infant physical activity and interactions

	Weekday				Weekend			
	beta	95%CI		*P* value	beta	95%CI		*P* value
Mother’s PA	0.107	0.072	0.143	0.000	−0.040	−0.088	0.009	0.107
Infant age (months)	0.627	0.313	0.942	0.000	0.936	0.626	1.246	0.000
Mother with infant (Yes)	−12.654	−15.415	−9.894	0.000	−20.193	−24.092	−16.294	0.000
Interaction Mother PA X Mother with infant	0.166	0.139	0.192	0.000	0.231	0.190	0.272	0.000
Infant in bed (No)	20.287	18.862	21.712	0.000	20.026	18.083	21.968	0.000
Mother in bed (No)	0.427	−1.157	2.011	0.597	−0.608	−2.804	1.588	0.588
Interaction Mother’s PA X Mother in bed	−0.037	− 0.062	−0.013	0.003	0.024	−0.004	0.053	0.094

All regressions are controlled for hour of the day, mother’s age, BMI, and duration apart from infant, maternal employment; and infant age, gender, BMI, developmental stage, and having a sibling

*PA* physical activity, *BMI* body mass index

**Fig. 1:**
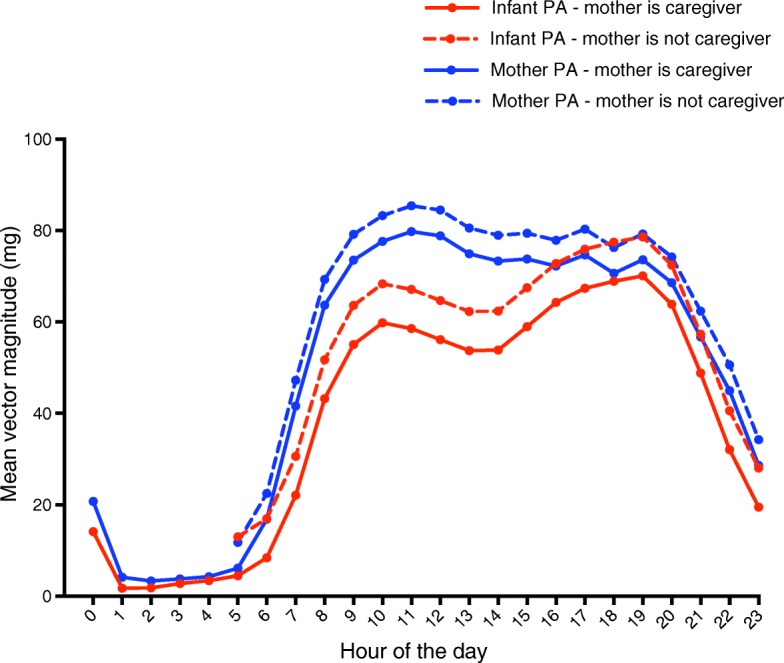
Diurnal physical activity patterns for mother and infant when mother is the caregiver (solid lines) compared to when mother is not the caregiver (dotted lines). Infant activity is corrected for infant age and gender, and for the interaction between hour of day and day of week, and interaction between hour of day and caregiver status of the mother. Mother activity is corrected for the interaction between hour of day and day of week, and interaction between hour of day and caregiver status

The diurnal distribution of the infant and maternal physical activity, stratified by mother’s caregiver status and week/weekend day, is presented in Fig. 1. By visual interpretation it is evident that in all instances, peaks of physical activity were higher when the mother was not the primary caregiver (1203 person-hours of data considered) compared to when the mother was the primary caregiver (18,851 person-hours of data considered) for each given hour of the day. This held true for both mother and infant physical activity levels.

When stratifying infants by age (rounded to the nearest month), and week/weekend day, while considering only time-not-in-bed, infant physical activity during the week was significantly higher when the mother was not the primary caregiver at all ages except for at 18- and 24-months, at which ages infant PA was higher when the mother was the caregiver (Table 3). During the weekend, infant physical activity was significantly higher when the mother was the primary caregiver at 6–12- and 24- months; but the opposite was true at 3- months. There was no difference in the overall relationship when stratifying by gender rather than age compared to the pooled sample; both boys and girls were significantly more active when their mother was not the primary caregiver on weekends as well as during weekdays. However, the strength of the differences between physical activity levels according to caregiver status was stronger in girls than boys (*p* < 0.01 on both weekdays and weekend days for girls; and *p* = 0.002 on weekends and *p* = 0.03 on weekdays for boys).

**Table 3:** Average infant physical activity stratified by age, maternal caregiver status, and week or weekend day. All values are mean (standard error of the mean, SEM)

	Weekday			Weekend		
	Mother is caregiver	Mother is not caregiver	*p*-value	Mother is caregiver	Mother is not caregiver	*p*-value
3-months (*n* = 31)	40.4 (0.5)	47.2 (2.0)	< 0.01	41.9 (0.6)	34.4 (2.3)	0.02
6-months (*n* = 31)	51.2 (0.5)	65.4 (2.3)	< 0.01	54.5 (0.7)	66.6 (3.7)	< 0.01
12-months (*n* = 30)	59.6 (0.6)	66.3 (0.6)	< 0.01	59.8 (0.8)	66.3 (2.0)	< 0.01
18-months (*n* = 20)	72.7 (0.9)	69.5 (2.5)	< 0.01	73.5 (1.2)	74.6 (2.7)	0.88
24-months (*n* = 30)	78.1 (0.9)	68.2 (1.8)	< 0.01	79.8 (1.1)	85.9 (2.8)	0.01

## Discussion

This is the first study that we are aware of to have examined the diurnal relationships between maternal and infant objectively measured physical activity, specifically taking into account whether the mother was with her infant or not during each time period. Infant physical activity was consistently and strongly associated with their mother’s physical activity, yet this relationship was modified by whether the mother was the infant’s primary caregiver at the time, and was different on weekdays compared to weekend days. For both mother and infant, physical activity was significantly higher on weekend days compared to weekdays. Furthermore, when taking into account the time of the day, age of the infant, and whether it was a weekday or weekend day, both mother and infant physical activity was higher during periods when the mother was not the primary caregiver.

It is important to note that the majority of mothers did not report spending any time apart from their infant during the study period, and so for the majority of time periods analysed the mother and infant were together. The higher levels of physical activity observed on weekend days compared to weekdays has been reported in older children [41], but is a novel finding in this young age group. Recently, a study conducted in older children (aged 5 years) and their mothers showed differences in the associations between co-participation in specific activities and the child’s physical activity on weekdays compared to weekend days [42]; however mother and child moderate to vigorous physical activity was significantly correlated in both instances. The difference in direction of interactions observed between mother and infant physical activity on weekdays compared to weekend days in the current population is unique. On both weekdays and weekend days, the combined effect of mother’s physical activity in conjunction with the interactions between sleep status and caregiver status on infant physical activity levels was approximately the same, and was largely due to the interaction between mother’s physical activity and being the primary caregiver; yet on weekdays this effect was strengthened by the mother’s physical activity main effect while on weekend days the effect was attenuated by mothers physical activity main effect. These relationships suggest that mother’s habitual physical activity alone may not be as strongly associated with infant physical activity as is her physical activity when she is looking after her infant, particularly on the weekend. In other words, what the mother is doing while she is with her infant seems to be the factor most strongly associated with infant’s physical activity levels. It is thus possible that infant’s behaviours may be dependent on interactions with-, and opportunities provided by their mothers or caregivers, particularly in the younger infants. It is, of course, possible that the direction of these associations are the opposite, such that more active infants stimulate mothers to be more active while taking care of them. However, regardless of the direction of this association, mothers (caregivers) would need to be the target for intervention, and should be encouraged to be more active (while with the infant or alone), as well as to provide infants with opportunities to be active. This is an important finding since it highlights a target area for intervention, particularly since the majority of mothers in this sample appeared to be with their infants at all times, increasing the potential for impacting infant behaviours. According to these findings, if mothers are encouraged to be more physically active while with their infant, this could lead to higher infant activity levels.

Also worth considering is the fact that both mother and infant physically activity levels were found to be higher at time periods when they were not together i.e. when the mother was not the caregiver. Somewhat similar to this although in an older age group, Jago et al. reported that in a sample of 10–11 year old children and their parents, while physical activity levels were similar between children and parents, it did not seem that activities were being done at the same time [17]. These findings potentially provide some insight into how mothers are interacting with their infants. Possibly, when mothers are not looking after their infants they are able to move about more freely, or choose to take this time alone to participate in physically active pursuits. Qualitative work done on mothers of infants in Midwestern United States has shown that mothers reported that they purposefully chose to engage in active behaviours when alone as a form of ‘release’, and very few engaged in active behaviours with their infant [43]. It is likely (and largely confirmed by the data collected on employment status in this sample) that for the majority of the time when mothers were not the primary caregiver of their infant, they were at work. Higher activity levels may therefore have been accumulated through active transport, which is still highly prevalent in South Africa [44], or through physically demanding jobs. Conversely, when mothers were looking after their infants they may have been spending this time in sedentary behaviours (such as watching TV, resting, sitting with their child while feeding). Sedentary behaviour participation is high in South African women, particularly in the age group within which these mothers fall [45], and so it is possible that these mothers were spending the majority of their time sedentary. Parent and child sedentary behaviours and TV viewing have been shown to be strongly correlated with each other, in some cases more so than correlations between higher intensity activities [16, 17].

In the present study, the behavioural interaction between the mother and infant that was occurring when they were together also seemed to be associated with the infant accumulating less activity. This could be attributed to co-participation in low intensity activities, likely in conjunction with mothers not providing access or opportunities for infants to be active, or due to some kind of learnt behaviour or role modeling - which may be more likely in the older age groups. In fact, when stratifying by age, older infants (> 18 months) were actually more active when their mother was looking after them during the week. Thus, it is possible that older infants have more autonomy and that their behaviours are less associated with their mother’s activity levels, or that older infants are provided with more opportunities to be active (such as access to outdoor space) [46–48] regardless of what their mother is doing at the time. These considerations do not explain why infants are more active when their mother is not looking after them – presumably in these instances they are being looked after by another adult, and in Soweto this would often be another family member [49]. The reason for the mother’s presence specifically influencing infant activity levels in an adverse manner is not clear. It is possible that non-maternal caregivers provide infants more opportunity to be active, by restraining them less and allowing them to move more freely. This may be due to the caregiver having multiple children to look after at a time, potentially having other chores to do around the home, and thus not spending as much time holding or carrying the infant. In Soweto, women often look after multiple children (biological and non-biological) in the home at a time [32]. Twenty of the mothers in the current study reported that their infant was in the care of another family member when not in their personal care, and family members consisted of grandmothers, aunts or siblings. Therefore it is likely that infants who were not with their mother were being looked after in the vicinity of other infants or children, thus increasing their likelihood to play alone or with these children [25]. In the current study having siblings was significantly associated with infant physical activity, and it is likely that infants could be with their siblings when not with their mother, thus increasing their activity levels. The same may be true for caregiving on weekends, during which time the mother may be with her infant but may not be the only caregiver, in which case her activity may be less related to her infant’s activity. This provides a potential explanation for the attenuation of the relationship between mother and infant physical activity by mothers’ physical activity on weekends only. Some children were reported to be attending a crèche or pre-school (*n* = 4) or looked after by a nanny (*n* = 8), which could possibly allow for a more stimulating environment with more opportunity to be active than they would receive at home with their mother.

It is unclear why this relationship is only significant on weekdays, and some context around the types of activities infants participate in on different days of the week would be useful. When stratifying by gender, while there were no significant differences between boys and girls, the effects of caregiver status on physical activity was much weaker for boys. Studies have shown that male children tend to receive more support for physical activity than females, and that this support is associated with higher physical activity levels [10]. It may be that mothers and other caregivers are providing male infants with more opportunities to be active, thereby attenuating differences that are dependent on mother caregiver status. It has also been shown that mothers may have more influence on their daughter’s than their son’s physical activity levels; while fathers may have more influence on their son’s activity levels [10]. Since we did not measure father’s activity levels, we cannot explore this possibility; however in Soweto many fathers do not live with their children and therefore may not play as strong a role as is seen in other contexts [32] – in fact only one mother in the present study reported that the father of the infant was a caregiver during the study period, although this may have been interpreted as the specific tasks of feeding and nappy changing, and not neccesarily being present. It is possible that this study is showing some support for previous findings that mothers are not as likely to influence their sons’ physical activity levels as they are their daughters’ physical activity levels. Interventions should thus consider how best to target male infants’ activity levels, and should be aware that female infants may be more likely to be influenced by their mother’s behaviour.

An obvious limitation to this study is the lack of contextual information, which limits our understanding of the types of activities being done when mother and infant are together or apart, where these activities are being done, and who was interacting with the infant when they were not with their mother. Furthermore, it is possible that even when the mother was the primary caregiver, she was not directly interacting with her infant, thus complicating the interpretation of the correlation between mother and infant behaviours. In addition, the lack of contextual information means that we were unable to differentiate between infant accelerometer readings that were generated by self-initiated infant movements, compared to those which were generated by the mother or another caregiver moving the infant (i.e.: picking the infant up, rocking, changing etc). This is a limitation for any study attempting to objectively assess infant physical activity levels using accelerometers when movements are not always self-initiated, and further work is required in order to address this issue. A further methodological issue was the lack of information obtained on sleeping/napping during the day, which limits the interpretation of the results, particularly for younger infants. Some of these findings may also be context specific, and therefore cannot necessarily be extrapolated to a population of infants outside of a similar low-middle-income setting. We also did not obtain information on the mother’s level of education, which may have impacted on her interpretation of some of the questions. Lastly, due to the cross-sectional nature of the study, firm conclusions on causality cannot be drawn, and longitudinal studies and trials are required to further elucidate these relationships.

## Conclusions

In conclusion, this study has shown that infant physical activity levels were strongly associated with their mother’s activity levels particularly during the week, yet this relationship was stronger when mothers were being active while looking after their infant. Furthermore, both mother and infant were less active when together than when apart, and were less active on weekdays than on weekend days. These findings highlight potential targets for interventions aimed at increasing infant physical activity levels. Specifically, mothers should be encouraged to be active as much as possible when looking after their children, and to attempt to increase these levels during the week. In all instances, infants should be provided with as much opportunity to be active as possible.

## Abbreviations

BMI: Body mass index
WHO: World Health Organization
